# The Transcription Factor NFATp Plays a Key Role in Susceptibility to TB in Mice

**DOI:** 10.1371/journal.pone.0041427

**Published:** 2012-07-23

**Authors:** Laura E. Via, Alla V. Tsytsykova, Ricardo Rajsbaum, James V. Falvo, Anne E. Goldfeld

**Affiliations:** 1 Tuberculosis Research Section, Laboratory of Clinical Infectious Diseases, National Institute of Allergy and Infectious Disease, National Institutes of Health, Bethesda, Maryland, United States of America; 2 Program in Cellular and Molecular Medicine, Children's Hospital Boston and Immune Disease Institute, Harvard Medical School, Boston, Massachusetts, United States of America; University Paris Sud, France

## Abstract

In T cells, the transcription factor nuclear factor of activated T cells p (NFATp) is a key regulator of the cytokine genes tumor necrosis factor (TNF) and interferon-γ (IFN-γ). Here, we show that NFATp-deficient (NFATp^−/−^) mice have a dramatic and highly significant increase in mortality after *Mycobacterium tuberculosis* (MTb) infection as compared to mortality of control animals after MTb infection. Animals deficient in NFATp have significantly impaired levels of TNF and IFN-γ transcription and protein expression in naïve or total CD4^+^ T cells, but display wild-type levels of TNF mRNA or protein from MTb-stimulated dendritic cells (DC). The rapid mortality and disease severity observed in MTb-infected NFATp^−/−^ mice is associated with dysregulated production of TNF and IFN-γ in the lungs, as well as with increased levels of TNF, in their serum. Furthermore, global blocking of TNF production by injection of a TNF neutralizaing agent at 6 weeks, but not 12 weeks, post-MTb-infection further decreased the survival rate of both wild-type and NFATp^−/−^ mice, indicating an early role for TNF derived from cells from the monocyte lineage in containment of infection. These results thus demonstrate that NFATp plays a critical role in immune containment of TB disease *in vivo*, through the NFATp-dependent expression of TNF and IFN-γ in T cells.

## Introduction

*Mycobacterium tuberculosis* (MTb) is an intracellular bacterium that causes tuberculosis (TB) infection in humans. Since a healthy immune system can usually effectively keep the bacilli in check, ∼90% of infected humans do not develop active disease in their lifetime unless they are immunosuppressed by drugs or disease [1], [2], [3], [4], [5], [6], [7]. A strong multicellular immune response is required to maintain TB in its latent state after infection [1], [4], [6], [8], [9], [10]. In the case of a weakened immune system, latent TB reactivates into active disease with a significant increase in frequency. HIV infection is a significant risk factor for the development of active TB [11], [12]. By compromising the immune system, HIV greatly facilitates the activation of latent TB. TB remains the world's deadliest infectious killer next to HIV, claiming approximately 1.5 million human lives annually, and remains the largest cause of death in HIV co-infected individuals [6], [13], [14], [15], [16].

Resistance to TB involves macrophages [2], [4], dendritic cells (DC) [17], [18], and T cells [18], and multiple studies in humans and mice have shown that maintenance of TB latency requires a Th1 T cell immune response and IFN-γ, which is a signature cytokine of this response. Also required are the participation of alveolar macrophages and T lymphocytes, and the production of cytokines such as IL-2, IL-12, IL-18, and TNF, as well as chemokines such as RANTES, MCP-1, MIP-1α, and IL-8, which play an important role in the migration of different cell populations to the site of granuloma formation [6], [9], [10], [19]. Humans with genetic deficiencies in IFN-γ as well as IL-12, which is produced by the monocyte/macrophage lineage, and/or their receptors are particularly susceptible to uncontrollable infection by MTb and by otherwise non-pathogenic strains of atypical mycobacteria and by *Mycobacterium bovis* bacillus Calmette-Guerin (BCG) [5], [20], [21], [22], [23]. Consistent with these observations, mice deficient in the transcription factor T-bet, which is required for IFN-γ production and establishing a general program of Th1 gene expression [24], [25], are particularly susceptible to MTb infection, which is correlated with relatively decreased IFN-γ production as compared to wild type mice [26].

TNF is produced by a number of cell types, including T cells, B cells, macrophages, and DCs [27], [28]. Regulation of TNF gene transcription is inducer- and cell type-specific and invovles the formation of higher-order enhancer complexes, or enhanceosomes, at the TNF promoter [27], [29], [30], [31], [32], [33], as well as distal regulatory sequences [27], [34], [35]. TNF plays a critical protective role in the innate and adaptive response to tuberculosis in humans and mice [36], [37]. For example, active TB was observed to develop at a high frequency in individuals latently infected with TB who were treated with a broadly neutralizing antibody against TNF for Crohn's disease or rheumatoid arthritis [38]. In mice, TNF has been shown to play a key role in the inhibition of mycobacterial growth *in vitro* [39] and in granuloma formation *in vivo* [40], [41], [42]. However, although the role of TNF in the pulmonary inflammatory response to MTb infection has been extensively demonstrated in murine models, the cellular source of TNF and the relative contribution of T cell- versus macrophage- or DC-derived TNF in protection against MTb and in clinical TB infection have not been defined.

The transcription factor NFATp (also known as NFAT1 and NFATc2) is a major regulator of T cell-derived cytokine gene expression [43], [44], [45], [46]. While NFATp plays a critical role in the transcription of the IFN-γ gene [45] and the TNF gene [47], [48] in T cells, it is not required for TNF transcription in the monocyte/macrophage lineage [33], [48]. This was strongly corroborated *in vivo* in mice by experiments that showed that NFATp^−/−^ mice were less susceptible to T cell superantigen-induced toxic shock, but were equally susceptible to lipopolysaccharide (LPS)-induced toxic shock [48]. Thus, the role of NFATp in the immune response to MTb infection, particularly in distinct cell types, presents a question of considerable interest that is tractable in the mouse model.

Here, we show that NFATp^−/−^ mice have a dramatic increase in mortality after MTb infection relative to wild-type mice (mean survival of 119 days versus 205 days, p = 0.0042), and that they had an MTb burden in their lungs approximately three orders of magnitude higher at the symptomatic endpoint relative to the lungs of wild-type mice at the time of sacrifice (8.6×10^8^ CFU/lung versus 8×10^5^ CFU/lung). We show that in the lungs of MTb-infected NFATp^−/−^ mice, there is a significantly decreased level of IFN-γ mRNA, while there in an initial increase of TNF mRNA expression that correlates with increased serum levels of TNF protein. Using *in vitro* analysis, we show that transcription and protein expression of TNF or IFN-γ from naïve or total CD4^+^ T cells was significantly impaired in NFATp^−/−^ mice, while TNF transcription and protein expression from DCs stimulated by TB was identical in NFATp^−/−^ and in wild-type mice. Furthemore, we show that blocking TNF with the pharmacological neutralizing agent Enbrel has a deleterious impact on survival of wild-type and NFATp^−/−^ mice when administered 6 weeks rather than 12 weeks following MTb infection, with a greater impact upon NFATp^−/−^ mice, indicating an early role for TNF derived from DC and monocytic cells in susceptibility to TB. Taken together, these results indicate that through its key role in regulating TNF and IFN-γ, NFATp controls a major checkpoint in T cell cytokine expression required for control of MTb infection in the mouse.

## Materials and Methods

### Mice

BALB/c NFATp^+/−^ mice (a generous gift from L.H. Glimcher, Harvard School of Public Health, Boston, MA) were bred and NFATp^−/−^ and wild-type (WT) littermates were genotyped by PCR as previously described [43] until a colony of NFATp^−/−^ was established. NFATp^−/−^ and WT (BALB/c) mice were harem-bred and maintained in pathogen-free conditions. Mice were used for experiments including infection at the age of 6–7 weeks. Their care was in strict accordance with NIAID and NIMR institutional guidelines, and all efforts were made to minimize suffering.

### Bacteria and infection

MTb (HN878 clinical strain) was grown in 7H9 broth supplemented as recommended by the manufacturer (Becton Dickinson, Sparks MD). All animal infection with aerosolized MTb was performed as previously described [49], [50] under BSL3 conditions in the animal facilities at NIAID. Enbrel (Wyeth Pharmaceuticals) and human isotype control antibody IgG1 (R&D Systems) were diluted in PBS and given intraperitoneally twice a week at 0.01 mg/mouse/dose, mimicking the physiological human dose (0.4 mg/kg) administered for rheumatoid arthritis. Experiments to determine differences in survival time were repeated at least twice.

### Necropsy and Histology

Mice were sacrificed at described time points and the lungs, spleen, and any observed lymph nodes were removed aseptically and divided into portions for bacterial load determination, leukocyte cell typing and enumeration, RNA preparation, and histology. Histology tissues were fixed in 10% neutral buffered Formalin. Tissues were embedded in paraffin and 5–10 µm sections were cut and stained with H&E and Ziehl-Neelsen acid-fast stain to localize the acid fast MTb.

### FACS analysis of cell surface markers

Age-matched NFATp^−/−^ and BALB/c mice (12 each) were infected with 30.3±12 (SD) colony forming units of MTb strain HN878 per mouse. Lung cells were prepared from naive and infected mice at 2, 4, and 6 weeks post infection by crushing and pressing though a cell strainer (Becton Dickinson Labware, Lincoln Park NJ), lysing the erythrocytes with NH_4_CL-tris, and washing twice with DMEM without phenol red and resuspended to 2×10^6^ per mL in PBS containing 0.1% bovine serum albumin and 20% mouse serum. Cells from 2–4 mice were pooled if the number of viable cells recovered per mouse was less than 1×10^6^ and microspheres (Caltag laboratories, Burlingame CA) were added in order to determine the absolute cell number. Pooling was required for naïve mice at all time points and for the 2 and 4 week time points. The cells were stained for 30 min with antibodies (0.2 µg/10^6^ cells) against CD3 (clone 17A2, cy chrome) and CD11b (M1/70, FITC), and CD45 (clone 30-F11, R-PE), or CD8a (53-6.7, FITC), CD4 (clone RM4-5, cy chrome), and CD45 (clone 30-F11, R-PE) (BD Biosciences Pharmingen) in PBS containing 0.1% bovine serum albumin and 20% mouse serum. The cells were fixed with fixation medium (Caltag Laboratories) for 12 to 16 hours and analyzed by Fluorescence-activated cytometry using software provided by with the instrument (BD Immunocytometry Systems, San Jose, CA). The experiment was repeated with an additional set of infected mice since more mice were required per experiment than anticipated and the results were pooled.

### Isolation of murine primary T cells

For T cell purification, spleen cell suspensions from naïve (uninfected) mice were first depleted of APCs and CD8^+^ T cells using anti-B220, anti-CD8, and anti-CD11b antibodies. Total CD4^+^ T cells were purified by sorting for CD4^+^ and naïve T cells were purified by sorting for CD4^+^CD62L^+^CD45RB^high^ (98%) using a MoFlo flow cytometer (DakoCytomation). Cells were stimulated *in vitro* with anti-CD3/CD28 as described [51], [52].

### Cytokine measurement by intracellular cytokine staining (ICS)

Th1 and Th2 CD4^+^ T cell subsets were prepared by polarizing FACS-purified naive CD4^+^ T cells from NFATp^−/−^ or WT (BALB/c) mice in the presence of IL-12 and anti-IL4 (Th1 conditions) or IL-4 and anti-IFN-γ (Th2 conditions) for six days as previously described [51], [52]. Cells were then stimulated with a combination of anti-CD3 and anti-CD28 antibodies for 4 h in the presence of brefeldin A (10 mg/ml) for the last 2 h, then divided into two pools for double staining with IL-4 and IFN-γ or TNF and IFN-γ for analysis by ICS as described elsewhere [34], [51].

### Generation of BM-DC

Bone marrow (BM) cells were isolated by flushing murine femurs and tibia with culture medium and differentiated into dendritic cells (DC) in the presence of GM-CSF (50 ng/ml; a gift from DNAX Research Institute).

### Real-time quantitative PCR

cDNA was synthesized and analyzed by real-time PCR using specific oligonucleotides as described previously [51], [52]. Target gene mRNA expression was quantified using SYBR green (Applied Biosystems) and normalized to the ubiquitin mRNA levels.

### Cytokine ELISA

Blood serum from infected mice and supernatants from *in vitro* cultures of NFATp^−/−^ and WT primary cells were harvested at indicated time points and TNF and IFN-γ secretion was assessed by ELISA using mouse TNF and mouse IFN-γ Kits (R&D Systems).

### Statistics

GraphPad Prism (GraphPad Software, La Jolla California USA, www.graphpad.com) was used to prepare Kaplan Meier survival curves, calculate Log-rank test, and median time to morbidity. A students *t*-test was used to determine the difference in ELISA values between treatment and genotyped groups.

## Results

### NFATp-deficient mice are more susceptible to MTb infection

We infected 20 WT and 16 NFATp^−/−^ mice with the aerosolized clinical MTb strain HN878 and compared the survival of the animals. We note that five random mice were sacrificed after initial infection to demonstrate that the appropriate range (149 out of a range of 20–200) of MTb colony-forming units (CFU) had been delivered. Infected mice were then placed in a time-to-death experiment and were regularly weighed and bled. Any animals reaching the endpoint established for TB infection according to the protocol guidelines were sacrificed and at that time their lung, spleen, and serum were collected for CFU determination, histological examination, and cytokine analysis. Since the NFATp^−/−^ mice were bred on the relatively TB-resistant BALB/c background [43], [53], BALB/c mice were employed as the control strain, and the MTb strain HN878, which has previously been shown to cause accelerated death in murine TB models [49], [54], was used to infect the mice.

**Figure 1:**
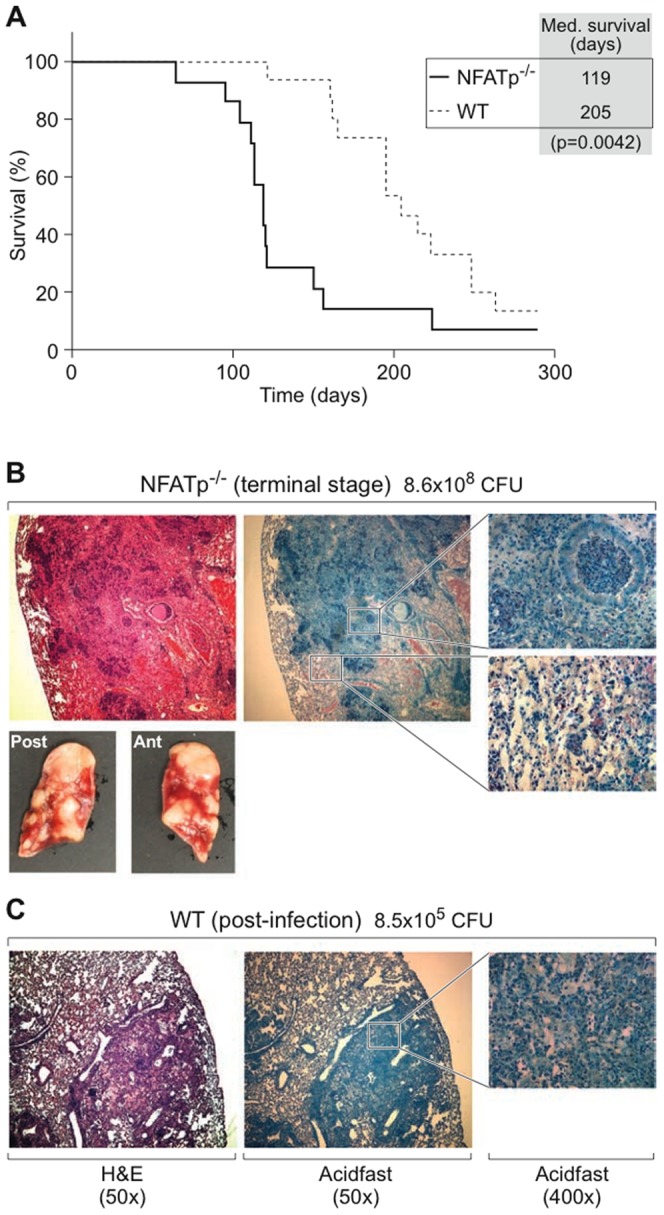
Increased susceptibility of NFATp^−/−^ mice to aerosolized *M. tuberculosis*. **A.** Survival of BALB/c (WT) vs. NFATp^−/−^ mice after infection with MTb. 20 WT and 16 NFATp^−/−^ mice were infected with the aerosolized clinical MTb strain HN878 (∼149 CFU) and monitored for survival. **B** and **C.** Lung pathology. (**B**) NFATp^−/−^ and (**C**) WT mice were infected with MTb, and lungs taken from premortal NFATp^−/−^ mice and from the WT mice sacrificed at the same time were fixed in 10% neutral buffered Formalin. Fixed tissues were embedded in paraffin, and 6–10 µm sections were H&E or Ziehl-Neelsen acid-fast stained as indicated.

As shown in Figure 1A, NFATp^−/−^ mice had a dramatically decreased median TB survival rate (119 days) as compared to WT mice (205 days, p = 0.0042). Consistent with this finding, the CFU count in the lungs of NFATp^−/−^ animals was approximately three orders of magnitude higher (8.6×10^8^ CFU/lung) at the symptomatic endpoint as compared to the CFU count in lungs of WT mice (8×10^5^ CFU/lung) sacrificed at the same time. Furthermore, at this morbid stage, the lungs of NFATp^−/−^ mice revealed rampant infection with bacilli outside of granulomas and massive cellular infiltration of the airways (Figure 1B). By contrast, lungs from WT animals at the same time post-infection showed lymphocytic infiltration without exuberant bacterial growth or evidence of vital tissue damage (Figure 1C), indicating the inability of the NFATp*^−/−^* mice to maintain control of the infection.

#### Dysregulation of IFN-γ and TNF mRNA levels in the lungs of MTb-infected NFATp^−/−^ mice

Animals deficient in IFN-γ and TNF or their receptors are highly susceptible to TB [55], [56]; however, the cellular source of the TNF that is important in TB containment has not been identified. Given that transcription of the Th1 cytokine IFN-γ and T cell-derived TNF have previously been shown to be NFATp-dependent [34], [45], [48], we next examined lungs from MTb-infected WT and NFATp-deficient mice and determined the levels of IFN-γ and TNF mRNA. For comparison, we also evaluated mRNA levels of the canonical Th2 cytokine gene IL-4 in NFATp^−/−^ and wild-type mice, since mice deficient in IL-4, including mice of the BALB/c background, do not display decreased survival following MTb infection [56], [57], [58]. We harvested lungs from two to three WT and two to three NFATp-deficient mice that were sacrificed between two and ten weeks after TB infection, then purified total RNA, and performed real-time PCR using IFN-γ-, TNF-, or IL-4-specific primers.

**Figure 2:**
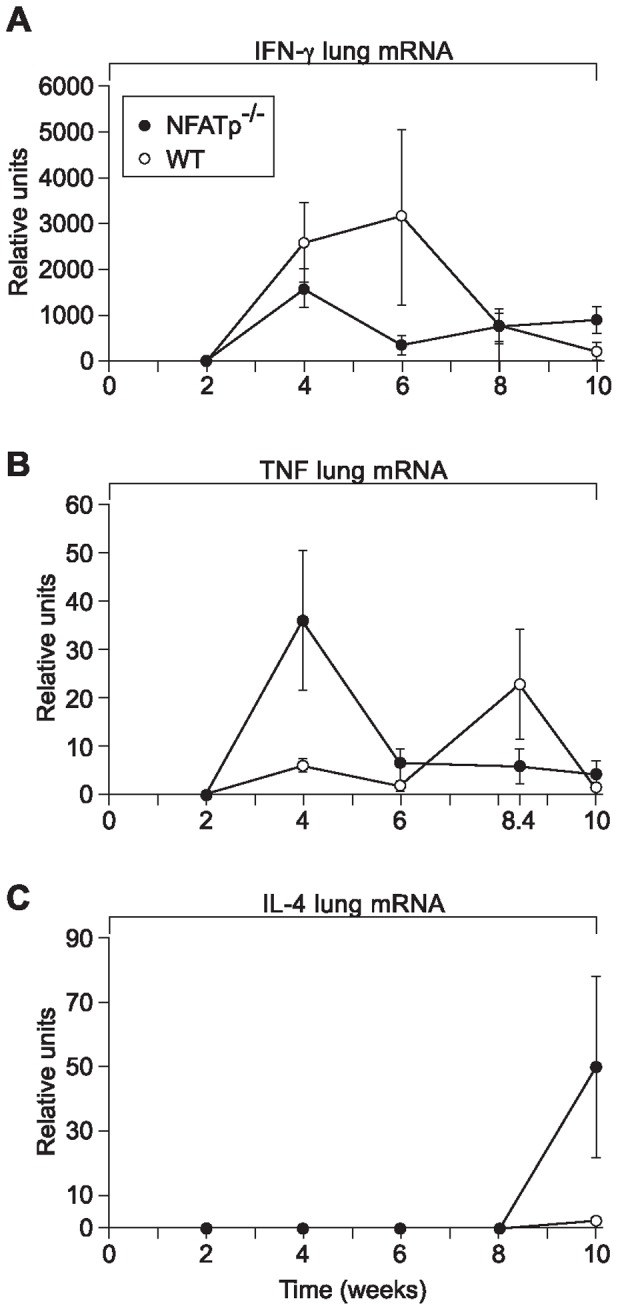
Distinct dynamics of cytokine mRNA expression in the lungs of MTb-infected NFATp^−/−^ and WT mice. NFATp^−/−^ and WT mice were infected with MTb and total RNA was purified from the lungs at 2, 4, 6, 8 (8.4), and 10 weeks post-infection. (**A**) IFN-γ, (**B**) TNF, and (**C**) IL-4 mRNA was measured by real-time PCR and values were normalized to ubiquitin mRNA. Error bars indicate mean of 3 samples ±SD.

Levels of IFN-γ mRNA in the lungs of WT and NFATp^−/−^ animals showed the most marked difference at 6 weeks post-TB infection, at which point the IFN-γ mRNA levels were significantly lower in NFATp^−/−^ mice, following relatively reduced levels of IFN-γ mRNA in NFATp^−/−^ mice observed at 4 weeks post-TB infection (Figure 2A). Given the necessity of an intact IFN-γ signaling pathway to control the early stages of murine TB infection [59], these data are consistent with the enhanced mortality of the NFATp^−/−^ mice. By contrast, TNF mRNA levels in the lungs of NFATp^−/−^ mice markedly peaked 4 weeks post-TB infection, becoming significantly higher than the corresponding levels in WT mice. By 8 weeks post-infection, however, WT mice TNF mRNA levels were significantly higher than those observed in NFATp^−/−^ mice (Figure 2B). This is consistent with enhanced TNF levels secondary to a dramatic inflammatory response derived from a non-T cell source. In addition, this dysregulated immune response evident in the lungs of NFATp^−/−^ mice is also reflected in the expression of IL-4. Levels of IL-4 mRNA in the lungs were very low up to 8 weeks post-TB infection regardless of the presence of NFATp (Figure 2C), consistent with previous studies in WT mice [56], [58]. However, NFATp^−/−^ mice showed a marked increase in IL-4 mRNA in the lungs at 10 weeks post-MTb infection (Figure 2C). This is reminiscent of earlier studies of splenocytes from this NFATp^−/−^ mouse strain in which, despite an initial defect in early IL-4 transcription, increased production of IL-4 was observed over the course of the immune response, coinciding with enhanced Th2 development [43].

**Figure 3:**
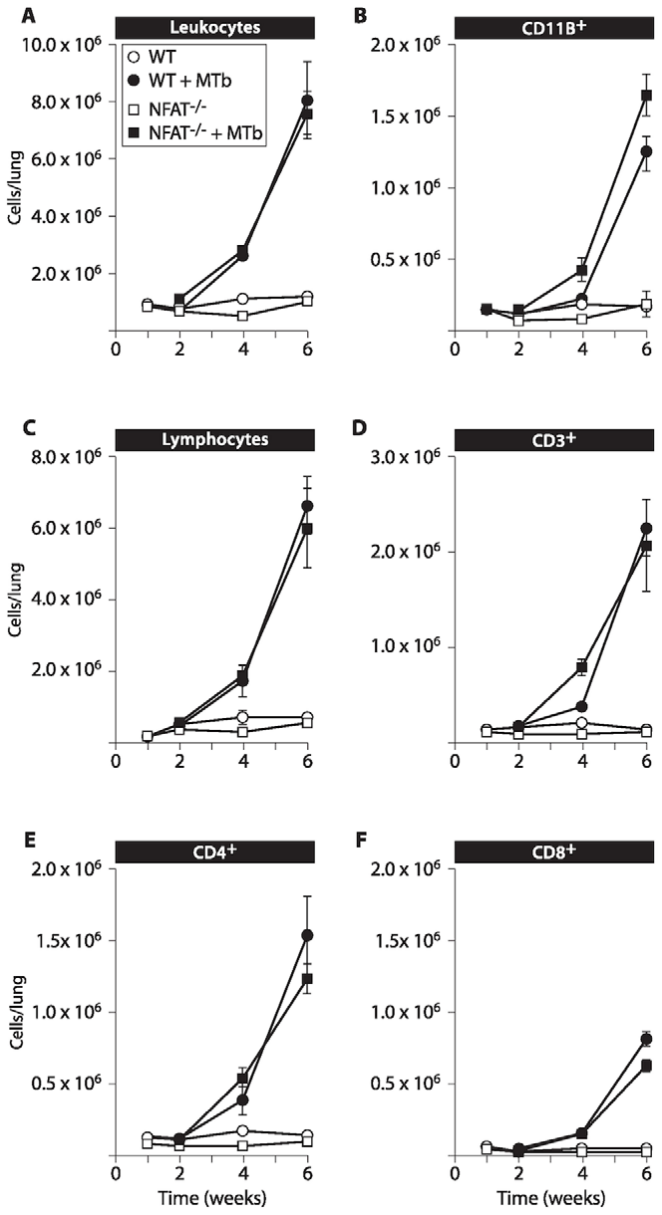
Infiltration of leukocytes into the lungs after MTb infection is similar in NFATp^−/−^ and wild-type mice. Age-matched NFATp^−/−^ and BALB/c (WT) mice were infected with aerosolized HN878 MTb strain at ∼30 CFU/mouse as described [50]. Lung cell samples from MTb-infected WT (•) and NFATp^−/−^ (▪) mice were obtained at 2, 4, and 6 weeks post-infection, and from uninfected WT (○) and NFATp^−/−^ (□) mice at times corresponding to 1, 2, 4, and 6 weeks post-infection, and the number of cells per lung for each indicated cell population (mean of 3 to 4 samples ±SD) was determined by FACS for samples at the indicated time points (**A–F**). The number of cells recruited to the lung during the course of MTb infection was determined for total leukocytes (**A**), CD11b^+^ cells, i.e., monocytes (**B**), total lymphocytes (**C**), CD3^+^ T cells (**D**), CD4^+^ T cells (**E**) and CD8^+^ T cells (**F**).

In order to rule out that the aberrant cytokine expression was secondary to perturbed leukocyte recruitment to the lungs in the NFATp^−/−^ mice, we next analysed the cell types present in the lungs of age-matched uninfected and MTb-infected NFATp^−/−^ and WT mice over the course of the first 6 weeks post-infection. As shown in Figure 3, the timing and number of recruitment of total leukocytes in the lungs of the NFATp^−/−^ and WT mice were similar (Figure 3A), with only a modest increase the number of CD11b^+^ cells (Figure 3B), indicative of the monocyte lineage, recruited to the lungs of NFATp^−/−^ mice relative to WT mice (1.65×10^6^±1.5×10^5^ vs. 1.25×10^6^±1.1×10^5^ cells, respectively, at 6 weeks post-MTb infection). The timing and number of recruitment of total lymphocytes (Figure 3C), CD3^+^ cells (Figure 3D), and CD4^+^ cells (Figure 3E) were also similar in NFATp^−/−^ and WT mice, with a slight increase in the number of CD8^+^ cells (Figure 3F) recruited to the lungs of WT mice relative to NFATp^−/−^ mice (8.06×10^5^±8.2×10^4^ cells vs. 6.3×10^5^±5.1×10^4^, respectively, at 6 weeks post-MTb infection). Thus, the observed dysregulation of IFN-γ or TNF mRNA in the lungs of NFATp^−/−^ mice was not correlated with differential recruitment of different immune cell populations to the lung. Taken together, these results demonstrate that production of IFN-γ and TNF are dysregulated in the lungs of NFATp-deficient mice following MTb infection, particularly within the first six weeks.

#### Serum TNF levels are increased in NFATp-deficient and wild-type mice during MTb infection and correlated with disease severity

**Figure 4:**
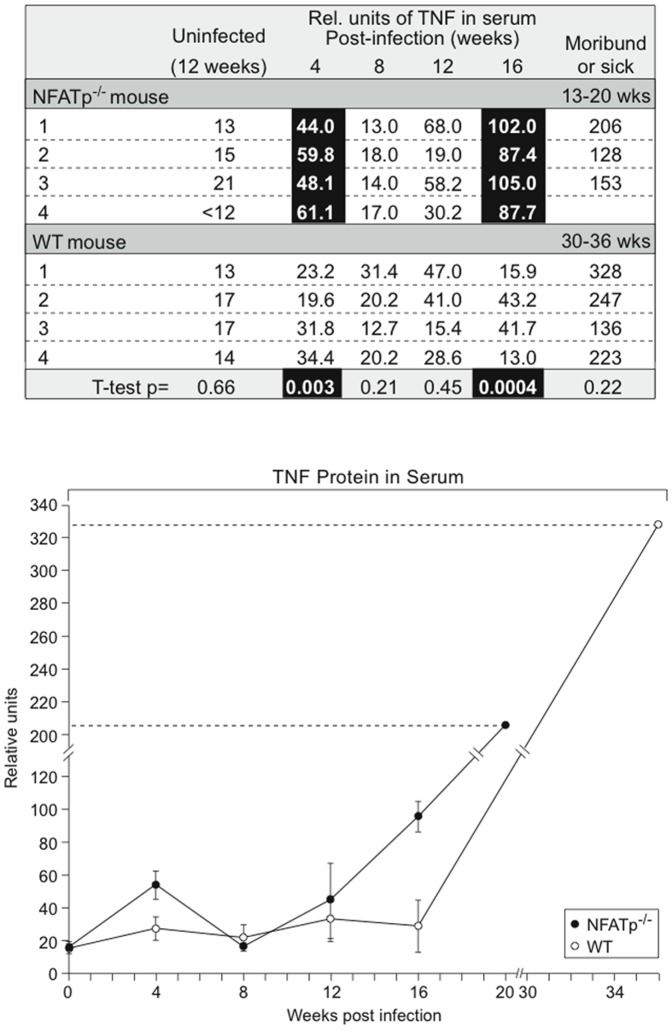
Correlation of serum TNF protein levels with MTb disease progression in WT and NFATp^−/−^ mice. WT and NFATp^−/−^ mice were infected with the aerosolized clinical MTb strain HN878 at ∼100 CFU/mouse as described [50]. Four random mice were bled at four-week intervals after initial infection and serum levels of TNF were measured by ELISA.

As shown in Figure 4, significantly higher levels of circulating TNF were detected in the serum of NFATp−/− mice at 4 weeks after infection, concordant with the increased TNF mRNA levels in NFATp−/− lungs at this time point, and they were again elevated at 16 weeks post-infection when the animals began to show clinical signs of severe disease. By contrast, at 16 weeks after infection, WT animals still appeared healthy and maintained lower TNF levels (Figure 4). Notably, near their respective median times of death (in NFATp−/− (13 to 20 weeks) or WT (30–36 weeks)), when CFU in the lungs were equivalent in the two groups of mice, there were no significant differences in TNF levels, which were elevated in both groups (Figure 4). Thus, increased serum levels of TNF were correlated with the severity of disease post-infection in both NFATp−/− mice and WT mice, and the higher TNF levels in NFATp−/− mice were consistent with their more rapid disease progression and mortality.

#### Loss of NFATp does not decrease TNF production by BM-DC but does decrease TNF and IFN-γ production by CD4^+^ T cells in vitro

**Figure 5:**
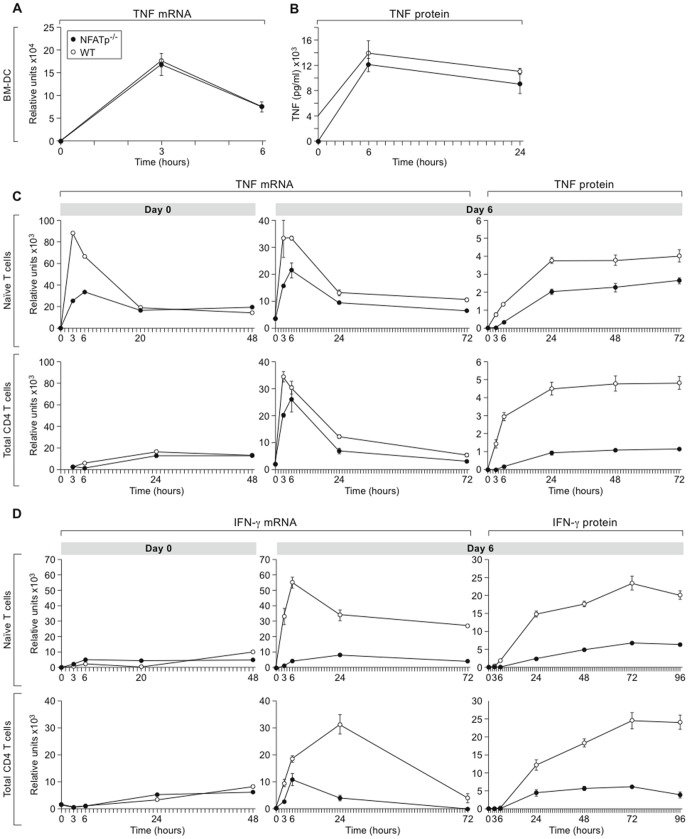
Varying NFATp requirement for cytokine expression in different primary cell populations. **A and B.** TNF expression in BM-derived dendritic cells is not NFATp-dependent. Primary BM-DC were derived from WT and NFATp^−/−^ mice. After 7 days cells were stimulated with MTb lysates. At indicated time points the cells were lysed to prepare total RNA. (**A**) TNF mRNA was measured by real-time PCR. (**B**) TNF protein levels in the supernatants were measured by ELISA. **C and D.** TNF and IFN-γ expression in murine primary T cells is NFATp-dependent. Purified primary total CD4^+^ and naïve (CD4^+^CD62L^+^CD45RB^high^) T cells from uninfected WT and NFATp^−/−^ mice were stimulated immediately with anti-CD3/CD28 antibody and then left in culture in neutral conditions for another six days and re-stimulated with anti-CD3/CD28 antibodies. Aliquots of cells were harvested at different time points post-induction as indicated. Purified total RNA was analyzed by real-time PCR with TNF and IFN-γ-specific primers, and cellular supernatants were used to measure TNF and IFN-γ protein levels by ELISA.

Since TNF produced by T cells is dependent upon NFATp, the finding that NFATp^−/−^ mice were able to produce high levels of TNF during acute TB infection indicated that cells other than T cells were able to vigorously produce TNF in response to TB in an NFATp-independent manner. Given the central role that DC play in phagocytosis and antigen presentation during acute TB infection in lungs [60], [61] and the high levels of circulating TNF in these mice, we next investigated whether DC production of TNF was impacted by NFATp deletion. We derived BM-DC *in vitro* from WT and NFATp^−/−^ mice, stimulated them *in vitro* with a TB sonicate, and analyzed TNF mRNA and protein levels. Strikingly, the level and kinetics of TNF transcription (up to 6 hours) (Figure 5A) and protein production (up to 24 hours) (Figure 5B) are not impacted by NFATp deficiency in BM-DC.

By contrast, when we examined TNF gene expression in primary naïve and total CD4^+^ T cells from WT and NFATp^−/−^ mice activated with anti-CD3/CD28 antibodies, TNF mRNA levels were significantly lower in cells from NFATp^−/−^ animals in response to both first and second cellular stimulations as compared to their WT counterparts (Figure 5C). Consistent with our previous findings with bulk CD3^+^ T cells [48], these transcriptional differences in TNF were more pronounced at early time points (within 3 hours) post stimulation (Figure 5C). TNF protein levels were also lower in naïve and total CD4^+^ T cells at all time points measured, consistent with the RNA results (Figure 5C).

In the case of IFN-γ, undifferentiated populations of T cells, naïve and total CD4^+^ cells from both WT and NFATp^−/−^ mice, produced very low IFN-γ mRNA in response to anti-CD3/CD28 stimulation (Figure 5D) consistent with the fact that the major source of IFN-γ is a differentiated population of T cells (Th1). When these same T cell populations were stimulated again with the same antibodies after 6 days of culture in neutral conditions, IFN-γ production was observed in the cells from WT animals consistent with their spontaneous differentiation into Th1 cells. In these T cell populations we observed a pronounced difference in IFN-γ gene expression and protein production between WT and NFATp^−/−^ mice at all time points up to 72 and 96 hours, with the NFATp^−/−^ T cells producing significantly less IFN-γ mRNA and protein at all time points (Figure 5D). For comparison, a previous study showed that at day 14 post-BCG infection, mediastinal lymph node cells from NFATp^−/−^ mice stimulated with purified protein derivative (PPD) from MTb or with a combination of anti-CD3 and IL-2 secreted less IFN-γ, but not less IL-5, than WT cells, consistent with a reduced Th1 response [62].

**Figure 6:**
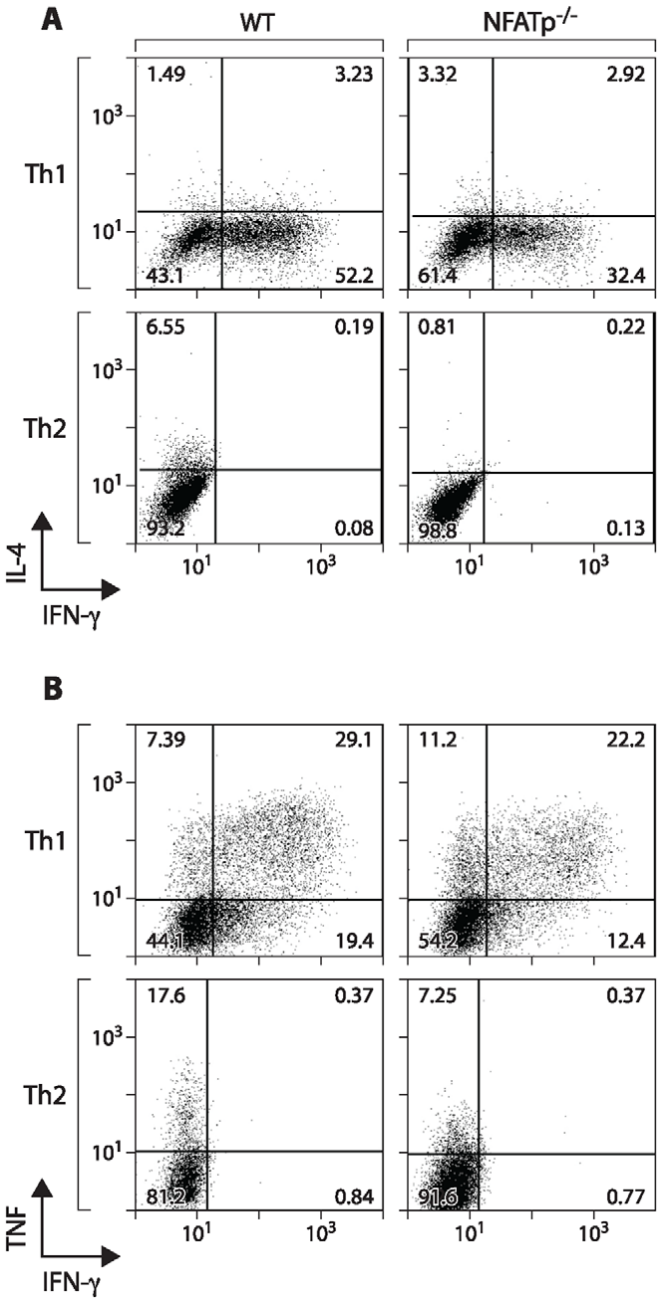
TNF, IFN-γ, and IL-4 production by CD4^+^ Th1 and Th2 cells from NFATp-deficient and WT mice. Primary CD4^+^ T cells from NFATp^−/−^ or WT mice were polarized into Th1 and Th2 populations for six days and subsequently stimulated with anti-CD3 plus anti-CD28 for 4 h before intracellular cytokine staining (ICS) for IL-4 and IFN-γ (**A**) or TNF and IFN-γ (**B**). NFATp-dependent production of IFN-γ protein by Th1 cells and IL-4 protein by Th2 cells is evident, while both Th1 and Th2 cells produce TNF protein with a more marked decrease in TNF expression in Th2 cells from NFATp^−/−^ mice.

As noted previously, while T cells from NFATp^−/−^ mice have an early defect in IL-4 gene expression following activation, they display a strong tendency to differentiate into Th2 cells [43], [44], [45], [63]. This can be ascribed to prolonged maintenance of IL-4 mRNA expression in NFATp^−/−^ T cells; indeed, T cells from NFATp^−/−^IL-4^−/−^ mice display wild-type levels of Th2 differentiation [44], [45]. We previously demonstrated, using intracellular cytokine staining and FACS analysis, that expression of TNF protein upon activation by anti-CD3/CD28 of both Th1 and Th2 cells is reduced in NFATp^−/−^ mice [34]. Using this approach, we thus next analyzed Th1 and Th2 cells from wild-type and NFATp^−/−^ mice for expression of TNF, IFN-γ, and IL-4 following activation with anti-CD3/CD28 (Figure 6). IFN-γ, as a canonical Th1 cytokine, is expressed almost exclusively in Th1 cells, and this expression is markedly reduced in Th1 cells from NFATp^−/−^ mice (Figures 6A and 6B). Similarly, IL-4 is predominantly expressed in WT Th2 cells, and its expression is strongly attenuated in NFATp^−/−^ Th2 cells (Figure 6A). In agreement with our previous results [34], TNF is expressed in Th1 and, to a lesser extent, Th2 cells, and the reduction of TNF expression in NFATp^−/−^ cells is much greater in Th2 cells relative to Th1 cells (Figure 6B). Thus, the decrease in IFN-γ and TNF mRNA and protein expression in activated NFATp^−/−^ CD4^+^ T cells is consistent with reduced IFN-γ expression in Th1 cells and reduced TNF expression in Th1 cells and, more dramatically, in Th2 cells.

Taken together, these experiments correlate impairment of T cell production of IFN-γ and TNF with the increased observed susceptibility of NFATp^−/−^ mice to TB. Moreover, they demonstrate that production of TNF from the DC compartment of NFATp^−/−^ mice in response to TB stimulation is not impaired and thus suggest that DC are a major source of NFATp-independent TNF transcription in this model. These experiments led us to next investigate whether the timing of TNF production in both the NFATp^−/−^ and WT mice was functionally important in the immune control of TB infection in these mice and/or the observed high TNF levels in lung and peripheral blood in the NFATp^−/−^ mice infected with TB were simply a marker of increased disease and earlier activation of DC or other monocytic cells where expression of TNF is not dependent upon NFATp.

#### Early blocking of total TNF signaling post-MTb infection leads to higher mortality in WT and NFATp^−/−^ mice

**Figure 7:**
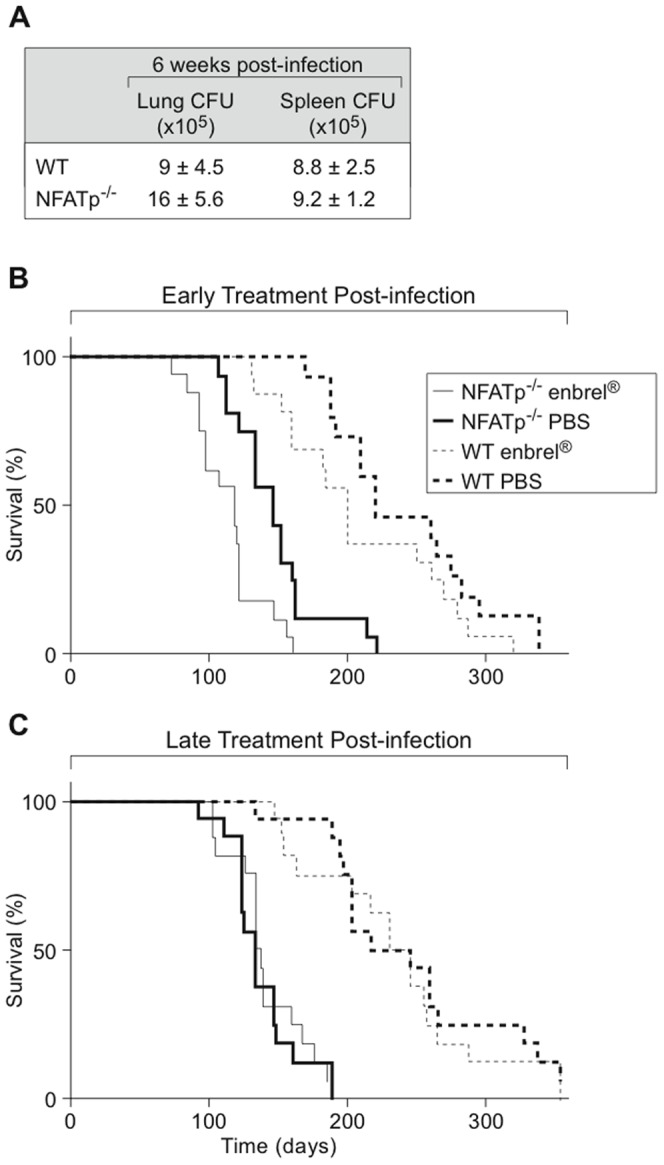
Time-dependent protective role of TNF during MTb infection. **A.** Comparable CFU counts in lungs and spleens from WT and NFATp^−/−^ mice infected with aerosolized MTb. **B** and **C.** NFATp^−/−^ mice and their BALB/c control mice (WT) were infected with aerosolized HN878 MTb strain at ∼100 CFU/mouse as described [50]. At 6 (**B**) and 12 (**C**) weeks post-infection, 16 WT and 16 NFATp^−/−^ were given twice-weekly intraperitoneal injections of Enbrel (0.4 mg/kg) and their survival was monitored. 16 WT and 16 NFATp^−/−^ mice served as controls and were injected at the same times with PBS.

To examine whether a further reduction in TNF levels by blocking TNF production in NFATp^−/−^ mice affected the mortality of these mice, we infected NFATp^−/−^ mice and age-matched WT mice with aerosolized HN878 in the presence or absence of the TNF neutralizing agent, etanercept (Enbrel), which contains the p75 TNF receptor binding domain attached to the human IgG1 hinge and Fc domains and reversibly binds both human and murine trimeric TNF [64]. At 6 and 12 weeks after infection, 16 WT and 16 NFATp^−/−^ mice were given twice-weekly intraperitoneal injections of Enbrel (0.4 mg/kg) and their survival was monitored. 16 WT and 16 NFATp^−/−^ mice served as controls and were injected at the same times with PBS. In separate experiments, both a human IgG1 antibody group and a PBS treated group were used as negative controls for the groups receiving Enbrel injections; irrelevant IgG treatment yielded results similar to PBS treatment (data not shown). In addition, uninfected NFATp^−/−^ mice were given intraperitoneal injections of Enbrel, PBS, or the IgG and no reduction in survival was noted. At 6 weeks post-infection, prior to treatment with Enbrel, CFU in the lung and spleen of NFATp^−/−^ and WT mice were approximately equivalent (Figure 7A).

As shown in Figure 7B, anti-TNF treatment administered early (beginning at 6 weeks post-infection) further significantly decreased NFATp^−/−^ survival by 20 to 28 days (median survival for NFATp^−/−^+PBS mice was 145 vs. 117 for NFATp^−/−^+Enbrel mice; p = 0.003). This result is consistent with blockage of TNF production leading to the increased mortality originating from monocytic cells such as DC that produce TNF in an NFATp-independent manner. Blocking TNF with Enbrel administration decreased the median survival of WT mice (200 vs. 220 days; p = 0.08), but not to the same extent as it did in the NFATp^−/−^ deficient mice. The limited effect of Enbrel treatment may be due to clearance of both Enbrel and the irrelevant antibody by the mouse immune system. Even when TNF was blocked in the WT mice, they still had significantly greater survival than the NFATp^−/−^ mice. These results are consistent with other factors such as IFN-γ playing a significant part in the enhanced mortality observed in NFATp^−/−^ mice (Figure 7B).

Strikingly, when anti-TNF treatment was administered late (beginning at 12 weeks post-infection), it had no significant effect on disease progression in either WT (median survival 225 days without vs. 232 days with Enbrel) or NFATp^−/−^ (median survival 130.5 days without vs. 132 days with Enbrel) mice (Figure 7C) indicating that TNF's role in host defense is most important early in infection. This result is consistent with the high TNF levels observed in the mice at the time of disease onset, which is reflective of the severity of disease. Thus, NFATp is essential for clearance of MTb even in the presence of high levels of TNF. Furthermore, the effect of NFATp is broader than its impact upon T cell-derived TNF production, consistent with our observation of the significant reduction of IFN-γ levels in the lungs and T cells of the NFATp^−/−^ mice.

## Discussion

Here, we have established a role for one of the NFAT family of transcription factors, NFATp, in protection against susceptibility to TB via its key role in regulating IFN-γ and T cell-derived TNF. The NFAT proteins regulate a wide variety of genes involved in the immune response, organ and tissue development, and T cell fate [46], [65], [66], [67], [68]. Studies employing gene disruption in mice have demonstrated that NFATp is specifically required for the expression of T cell-derived TNF [34], [43], [48] and IFN-γ [45]. IFN-γ is a central cytokine in the host Th1 response to MTb infection in humans as well as mice [6], [10], [28], [55], and patients with genetic disruptions in IFN-γ or the α chain of its receptor, IFN-γR, display increased susceptibility to mycobacterial infection [5]. Several studies in mice have shown that TNF is essential to control infection [37], [41], [56], [69], [70], [71] and in humans the treatment of autoimmune disease with a TNF blocking antibody results in the activation of latent TB infection [7], [38]. The cellular source of the TNF, however, was not previously known.

Extensive work performed in *in vivo* mouse models have demonstrated that anti-MTb immunity in mice is mediated predominantly by CD4^+^ Th1 cells with the aid of CD8^+^ T cells and involves IFN-γ signaling [1], [3], [56], [72], [73]. Most of these studies are based on the fact that mice that do not produce IFN-γ fail to inhibit MTb growth and rapidly succumb to the infection. Mechanistically, several studies have demonstrated that the impairment of the Th1 cytokine response and IFN-γ production leads to faulty anti-bacterial function in macrophages at sites of MTb infection [74], [75]. An elegant study using mice deficient in transcription factor T-bet, the master regulatory factor directing commitment to the Th1 lineage [24], [25], demonstrated that their increased susceptibility to TB was associated with decreased transcription of the T-bet-dependent IFN-γ gene [26]. The dramatically increased susceptibility to MTb infection that we observe in NFATp^−/−^ mice is consistent with their greatly impaired expression of IFN-γ in the lungs and CD4^+^ T cells, from Th1 cells in particular. Thus, our data from NFATp^−/−^ mice further link the defect of IFN-γ gene expression with increased susceptibility to MTb.

The regulation of IFN-γ gene expression by NFATp is part of a larger network of transcriptional regulation that directs differentiation of naïve T helper cells into Th1 and Th2 cells [46]. At early stages of T cell activation, NFATp binds to the promoters of both the IFN-γ and IL-4 genes, but this binding is later restricted to the IFN-γ promoter in Th1 cells and the IL-4 promoter in Th2 cells. Changes in NFAT binding are accompanied by alterations in chromatin organization as assayed by DNase hypersensitivity and histone acetylation at the IFN-γ and IL-4 loci [76], [77], [78]. The TNF locus also exhibits DNase hypersensitive sites and activation-dependent histone acetylation in T cells [27], [34], [79], [80], [81] as well as activation-dependent intrachromosomal interactions that involve NFATp-dependent promoter and enhancer regions [34], [82]. In the experiments presented here, we have shown that naïve or total CD4^+^ T cells from NFATp^−/−^ mice are impaired in their ability to produce IFN-γ and TNF mRNA and protein in response to MTb infection. We have further delineated the expression of these cytokines in CD4^+^ T helper cell subsets from NFATp^−/−^ mice: Th1-specific expression of IFN-γ is impaired and the expression of TNF in Th1 and Th2 cells is decreased, to a greater extent in Th2 cells.

Unlike IFN-γ, which is secreted by Th1 cells, NK cells, and NKT cells, TNF is expressed in a wide range of cell types, including T cells, B cells, DCs, macrophages, mast cells, and fibroblasts, and its cell type- and stimulus-specific regulation utilizes distinct signal transduction pathways, transcription factors, and coactivators [27]. We have previously shown that NFATp is a key activator in TNF gene regulation in T cells stimulated by calcium flux or via their antigen receptor, but not in monocytes and macrophages stimulated by LPS or MTb [29], [31], [32], [33], [34], [83]. In an *in vivo* toxic shock model, NFATp deficiency renders mice resistant to superantigen-induced T cell-mediated toxic shock without affecting their susceptibility to LPS-induced macrophage-mediated shock [48]. Here we have shown that in NFATp^−/−^ mice, while TNF mRNA and protein production is decreased in activated CD4^+^ T cells, BM-DC stimulated by MTb are not significantly affected in their ability to produce TNF mRNA and protein. This is consistent with earlier reports that transcriptional activation of the TNF gene promoter is NFAT-independent in a murine DC line [84], as is the case in a murine macrophage cell line [33], in contrast to NFAT-dependent TNF expression in T cells [29], [31], [34], [83], [85], [86], B cells [29], [87], [88], [89], and mast cells [84], [90]. The results presented here therefore provide further *in vivo* demonstration of the cell type-specific regulation of the TNF gene. In the context of previous data, these results reinforce the idea that NFAT-independent TNF expression in cells such as DCs and cells of the monocyte/macrophage lineage underlie the early peak of TNF expression we observe in the lungs and serum of NFATp^−/−^ mice.

Although T cell-specific expression of both TNF and IFN-γ was significantly impaired in the NFATp^−/−^ mice used in our study, the pattern of dysregulation of TNF expression in the lungs of NFATp^−/−^ mice was distinct from that of IFN-γ. While IFN-γ mRNA levels in the lungs of MTb-infected NFATp^−/−^ mice were decreased relative to WT mice up to 6 weeks post-TB infection, expression of TNF mRNA in the lungs in NFATp^−/−^ mice was transiently elevated during the acute phase of TB infection, reaching a peak 4 weeks post-infection and decreasing 6 weeks post-infection, while in WT mice the peak occurred approximately 8 weeks post-infection. Serum TNF levels were also higher at 4 weeks post-TB infection in NFATp^−/−^ mice relative to WT mice, and for both WT and NFATp^−/−^ mice serum TNF levels rose at later time points, starting at 16 weeks post-infection, in the premortal stage of TB. While there is thus a correlation between increased mortality of MTb-infected NFATp-deficient mice and increased levels of NFATp-independent TNF in the early stages of infection, for both NFATp^−/−^ and WT mice treatment with the TNF blocking agent Enbrel starting at 6 weeks post-infection significantly decreased survival, while injection at 12 weeks post-infection, when NFATp^−/−^ and WT mice had comparable and relatively low serum levels of TNF, did not affect survival, indicating that the contribution of TNF against susceptibility to TB occurs in the early, acute phase of MTb infection. Furthermore, the increase in mortality upon blocking TNF in the early phase of TB infection was more pronounced for NFATp^−/−^ mice, consistent with a protective effect of NFATp-dependent genes, particularly IFN-γ, in the host response to infection.

The T cell-specific defect in production of both IFN-γ and TNF in NFATp^−/−^ mice, and the lack of a robust Th1 immune response to MTb infection, underlie the increased susceptibility of NFATp^−/−^ mice to TB infection. Consistent with this idea, bacterial proliferation in the lungs of NFATp^−/−^ mice was markedly higher than in WT mice, and this increased bacterial burden, consistent with their relatively sicker state, could be expected to activate the monocytic compartment to produce high levels of TNF mRNA in lungs and protein in serum. This would likely include alveolar macrophages, which serve as the first line of defense from MTb pulmonary invasion [2]. While the cellular source and associated signal transduction pathways underlying the increase in TNF in the lungs of NFATp^−/−^ mice in the early phase of MTb infection remain to be elucidated, we have demonstrated that NFATp^−/−^ mice display a defect in IFN-γ expression in Th1 cells as well as in the lungs, while TNF expression is reduced in Th1 and, to a relatively greater extent, Th2 cells, but is not affected in DCs.

More broadly, this study has pinpointed a specific transcription factor involved in susceptibility to TB infection and has highlighted the important role of T cell-derived TNF and IFN-γ. The results presented here thus provide a framework for dissecting the cell type-specific role of NFATp and its upstream signal transduction pathways in the regulation of inflammatory response genes in general, and TNF in particular, involved in MTb infection.
